# Lipid-laden cells differentially distributed in the aging brain are functionally active and correspond to distinct phenotypes

**DOI:** 10.1038/srep23795

**Published:** 2016-03-31

**Authors:** Marilia Kimie Shimabukuro, Larissa Gutman Paranhos Langhi, Ingrid Cordeiro, José M. Brito, Claudia Maria de Castro Batista, Mark P. Mattson, Valeria de Mello Coelho

**Affiliations:** 1 Laboratory of Immunophysiology, Institute of Biomedical Sciences, Health Sciences Center, Federal University of Rio de Janeiro, Av. Carlos Chagas Filho 373, Rio de Janeiro, RJ, Brazil, 21941-902; 2 Laboratory of Cellular Proliferation and Differentiation, Institute of Biomedical Sciences, Health Sciences Center, Federal University of Rio de Janeiro, Av. Carlos Chagas Filho 373, Rio de Janeiro, RJ, Brazil, 21941-902; 3 Laboratory of Neurosciences, National Institute on Aging, National Institutes of Health, Baltimore, MD, USA, 21224-6825.

## Abstract

We characterized cerebral Oil Red O-positive lipid-laden cells (LLC) of aging mice evaluating their distribution, morphology, density, functional activities and inflammatory phenotype. We identified LLC in meningeal, cortical and neurogenic brain regions. The density of cerebral LLC increased with age. LLC presenting small lipid droplets were visualized adjacent to blood vessels or deeper in the brain cortical and striatal parenchyma of aging mice. LLC with larger droplets were asymmetrically distributed in the cerebral ventricle walls, mainly located in the lateral wall. We also found that LLC in the subventricular region co-expressed beclin-1 or LC3, markers for autophagosome or autophagolysosome formation, and perilipin (PLIN), a lipid droplet-associated protein, suggesting lipophagic activity. Some cerebral LLC exhibited β galactosidase activity indicating a senescence phenotype. Moreover, we detected production of the pro-inflammatory cytokine TNF-α in cortical PLIN^+^ LLC. Some cortical NeuN^+^ neurons, GFAP^+^ glia limitans astrocytes, Iba-1^+^ microglia and S100β^+^ ependymal cells expressed PLIN in the aging brain. Our findings suggest that cerebral LLC exhibit distinct cellular phenotypes and may participate in the age-associated neuroinflammatory processes.

Dyslipidemia is characterized by altered levels of lipids bound to lipoproteins in the circulation and occurs in association with oxidative stress and a systemic chronic basal inflammatory state, also known as “inflammaging”^1^,^2^. During aging and in metabolic disorders such as diabetes, lipid droplets, which are cytoplasmic non-polar lipids with lipophilic constitutive proteins called perilipins on their surface, accumulate in many organs including muscle and liver^3^,^4^. An association between the accumulation of lipid droplets and impaired autophagy, a cellular process where lysosomal enzymes degrade protein aggregates and damaged organelles, has been reported^5^. Although impaired autophagy may occur in brain cells during aging and neurodegenerative disorders^6^,^7^, it is not known if and in which brain regions accumulation of lipid-laden cells (LLC) occurs.

Several morphological changes occur in the brain architecture during aging. Morphometric analyses performed by neuroimaging showed that grey matter atrophy occurs in some regions of the human brain during aging including the prefrontal cortex, insula, anterior cingulate gyrus, superior temporal gyrus, precuneus and the inferior parietal lobule^8^. Additionally, aging induces shrinkage of most subcortical structures including the hippocampus and the striatum^9^,^10^. At the cellular level, there is a decrease of dendritic branching and spine density in neurons^11^, which contributes to disruption of synapses^12^. Regarding the white matter, myelin degeneration occurs in the frontal cerebral cortex during aging^13^,^14^, although the number of oligodendrocytes increases^12^,^15^ and these cells present swellings and dense inclusions in their cytoplasm processes and perikarya, possibly originating from the breakdown of degenerating myelin sheaths^16^.

Microglial cells are also affected during aging becoming less ramified and with shorter tortuous processes and rounded cytoplasmic swellings^17^,^18^. Functionally, microglia become hyperactive and secrete pro-inflammatory cytokines^19^, which are involved in the activation of astrocytes in the aging brain^20^. In fact, astrocytes in the aged brain exhibit enhanced calcium signaling, possibly in response to the local oxidative stress and basal inflammatory state^20^,^21^. With aging, astrocytes become hypertrophic and reactive presenting an increased expression of glial fibrillary acidic protein (GFAP) and vimentin intermediate filaments as well^22^,^23^. Furthermore, ependymal cells can co-express GFAP and S100β^24^ and their potential to accumulate lipid droplets has been recently shown in middle-aged mice^24^,^25^.

In contrast to knowledge of the roles of LLC in metabolic disorders and aging in liver and muscle cells, little is known of whether LLC accumulate in the brain in normal aging nor whether they play roles in age-related neuroinflammation. In the present study, we characterized cerebral LLC evaluating their distribution and morphology in the meningeal, cortical, subcortical and neurogenic areas during aging, and elucidated their functional activity and inflammation-related phenotypes.

## Results

### LLC are widely distributed and present distinct morphologies in the aging brain

**Figure 1:**
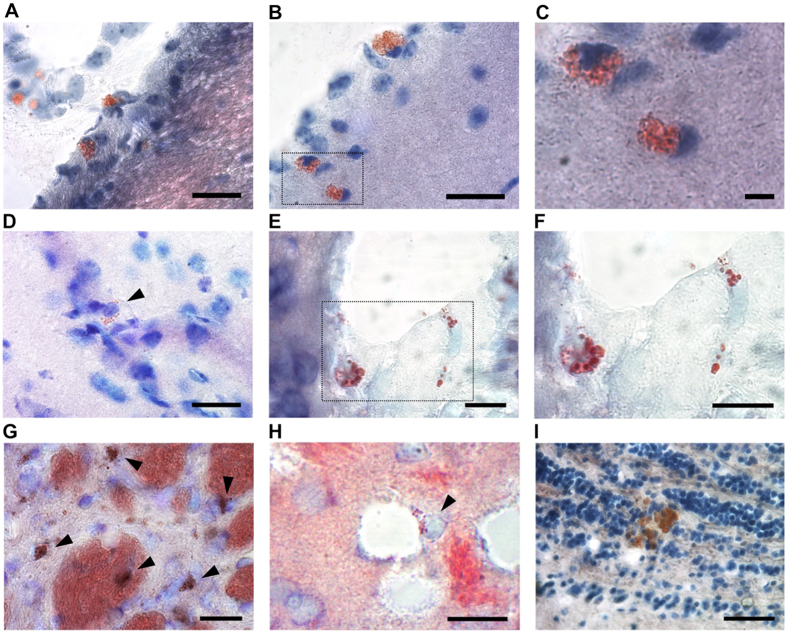
LLC are present in distinct brain regions of old mice. (**A**,**B**) Bright field micrographs show Oil Red O^+^ LLC in the pia mater (**A**) and in the pia mater and adjacent cortex (**B**). (**C**) Higher magnification of cortical LLC in area delineated in B. (**D**) Brain cortical LLC containing well-defined lipid droplets. (**E**) LLC associated with blood vessels in the cortex. (**F**) Higher magnification of delineated area in E. (**G**) Oil Red O^+^ LLC and fiber bundles in the striatum. (**H**) perivascular striatal LLC. (**I**) LLC in the olfactory bulb granular cell layer. Coronal brain tissue sections of aged mice were stained with Oil-red O and toluidine blue dyes. Arrowheads indicate Oil red O^+^ cells. Scale bars: 50 μm (**A,B,D,E,G,H,I**); 10 μm (**C,F**).

To analyze the distribution of LLC in the aged brain, we used Oil Red O staining in brain tissues of mice that were more than 18 months old. We first evaluated the presence of LLC in the pia mater layer of the meninges, which is composed of connective tissue mesenchymal cells derived from neural crest^26^. Indeed, mesenchymal cells are capable of differentiating into lipoblasts and may acquire a lipid-accumulating phenotype during aging in other organs^3^. In the pia mater, we found LLC with numerous small lipid droplets that we called lipid laden-multilocular cells (Fig. 1A). These cells were also located in the cortical region of the brain adjacent to the pia mater (Fig. 1B,C).

Analyzing the distribution of Oil Red O^+^ cells in deeper regions of the brain, we found LLC containing well-defined lipid droplets in their cytoplasm in the motor cortex, cingulate area, and somatosensory area (Fig. 1D). Some LLC with numerous small lipid droplets were also observed associated with blood vessels (Fig. 1E,F). In the striatum, which is the primary target of cortical input to the basal ganglia and is mainly involved in motor function, memory and cognition, numerous multilocular LLC were present including many in the parenchyma and some associated with myelinated axonal sheaths and perivascular areas (Fig. 1G,H). Furthermore, we identified a few multilocular LLC in the granular and glomerular layers of the olfactory bulb (Fig 1I).

**Figure 2:**
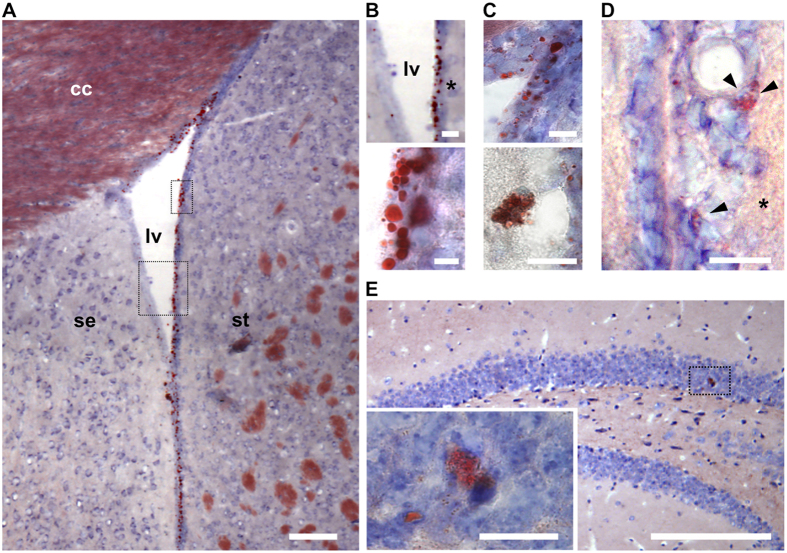
LLC are present in the lateral ventricle wall and hippocampal dentate gyrus neurogenic regions of old mice brains. **(A**) Representative bright field photomicrographs showing Oil-red O^+^ LLC along the lateral ventricular walls in the brain of an old mouse. (**B**) Higher magnification of delineated areas in A show differential distribution of Oil-Red O^+^ LLC between the medial and lateral walls (upper panel) and lipid droplets with variable sizes in cells of the lateral wall of the lateral ventricle (lower panel). (**C**) Oil-Red O^+^ LLC in the dorsal corner of the lateral ventricle (upper panel) or in association with blood vessels in the medial wall of the lateral ventricle (lower panel). (**D**) LLC in the lateral wall of the lateral ventricle subventricular area. (**E**) LLC in the hippocampal dentate gyrus. Inset shows higher magnification of delineated area with Oil Red O^+^ LLC. Coronal sections of the brain were stained with Oil-Red O and counterstained with toluidine blue. Asterisks indicate lateral ventricle lateral wall. Cc: corpus callosum; lv: lateral ventricle; se: septum; st: striatum. Scale bars: 100 μm (**A**,**E**); 20 μm (**B**,**C** upper panel; (**D**,**E**); 10 μm (**C** lower panel).

Oil Red O^+^ LLC were also located in the lateral ventricle walls and hippocampus, which include neurogenic niches (Fig. 2). In the lateral ventricle walls, we found that LLC were differentially distributed, being mainly observed in the lateral wall, which corresponds to the neurogenic striatal side, in relation to the medial or septal wall that exhibited few LLC (Fig. 2A,B). Ependymal cells in the lateral ventricle wall of old mice exhibited bigger lipid droplets than LLC observed in other regions, when we classified their diameters as either small (~0.5 μm) or large (~7 μm) (Fig. 2B,C). On the other hand, we observed scattered multilocular LLC close to blood vessels in the subventricular region of the medial wall (Fig. 2C) and lateral wall (Fig. 2D) of the lateral ventricles. In the hippocampus, only a few multilocular LLC were seen in the granule cell layer of the dentate gyrus (Fig. 2E).

Aiming to verify whether the lipid content in cerebral LLC corresponded to lipofuscin, which is cytoplasmic accumulation of lipid and protein debris resulting from extensive lysosome activity, we visualized brain tissue stained with Oil Red O under 488 nm UV light. Lipofuscin granules which are autofluorescent^27^ did not co-localize with Oil Red O^+^ lipid droplets in the brain tissue sections analyzed (Supplementary Fig. S1), indicating a distinct lipid content in cerebral LLC. However, we do not exclude the possibility that LLC can contain both lipid droplets and lipofuscin.

Collectively, our data indicate that LLC exhibit different morphologies and contain lipid droplets of distinct sizes; LLC increase in numbers and are widely distributed in the aging brain, including in the meningeal, cortical, subcortical and neurogenic areas.

### Increased density and differential distribution of LLC in the lateral ventricle walls and in the parenchymal versus perivascular areas of the cortex and striatum of aging mice

**Figure 3:**
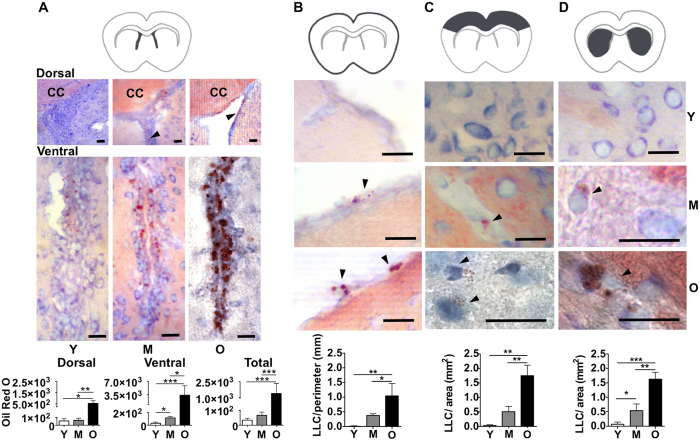
Quantification of Oil Red O^+^ LLC in the pia mater and distinct regions of the brain during aging. (**A**–**D**)(upper). Dark grey areas in the schema indicate brain regions analyzed in young (~3 month-old), middle age (~12 month-old) or old (>18 month-old) mice. (**A**–**D**) (middle). Representative photomicrographs of the dorsal and ventral subregions of the lateral ventricles; pia-mater; brain cortex; and striatum of young, middle-aged and old mice, as indicated in the figure. Black arrowheads point Oil Red O^+^ lipid droplets. Excepting 50 μm scale bar in the image of the lateral ventricle wall dorsal subregion of old mice, scale bars in all other images correspond to 20 μm. (**A**–**D**) (lower). Graphs show (**A**) Oil Red O intensity in lipid droplets per mm in dorsal or ventral subregions or in the total area of lateral ventricles;and the number of Oil Red O^+^ LLC per mm in (**B**) pia mater or per mm^2^ in (**C**) cortex and (**D**) striatum. Y, young; M, middle-aged; O, old mice. Data are presented as mean ± SEM (n = 3 mice per group). *p < 0.05, **p < 0.01, ***p < 0.001.

We performed *in situ* histomorphometry to evaluate the density of lipid droplets in cells located in the lateral ventricles walls as well as of LLC in the pia mater or defined areas of the cortex and striatum in brain tissue sections of mice, as schematized in Fig. 3.

Our data show that the density of lipid droplets in LLC in the lateral ventricle walls, including the dorsal and ventral subregions, increase with age (Fig. 3A). We also found a significant increase in the accumulation of lipids in the medial subregion of the lateral ventricles between young and old mice (Young: 4.3 ± 2.6; Old: 214.9 ± 11.8, P < 0.001). In addition, we verified that the density of Oil Red O^+^ staining in the lateral wall of the lateral ventricle is higher than in the medial wall, in both middle-aged and old mice (Table 1). However, this proportion decreases with age due to an increased number of LLC in the medial wall.

Regarding the quantification of LLC with numerous small lipid droplets, we observed a significant increase in the density of these cells in the pia mater, cortex and striatum with age progression (Fig. 3B–D). Further analyzing LLC in cortex and striatum regions, we found that the frequency of perivascular LLC (BV-LLC) was higher than LLC deeper in the parenchyma (P-LLC), in both middle-aged and old animals (Table 2). The ratio between BV- LLC and P-LLC in the cortex and striatum regions decreased between middle-aged and old mice, which was associated with an increase of P-LLC with age (Table 2).

These results show that the density of LLC increases in the pia mater and in all brain regions analyzed. Moreover, LLC exhibit a differential distribution between the lateral and medial walls of the lateral ventricles as well as between the parenchymal and perivascular areas of the cortex and striatum of aged mice.

### LLC exhibit autophagosome formation, senescence associated β-galactosidase activity, and produce the pro-inflammatory cytokine TNF-α

**Figure 4:**
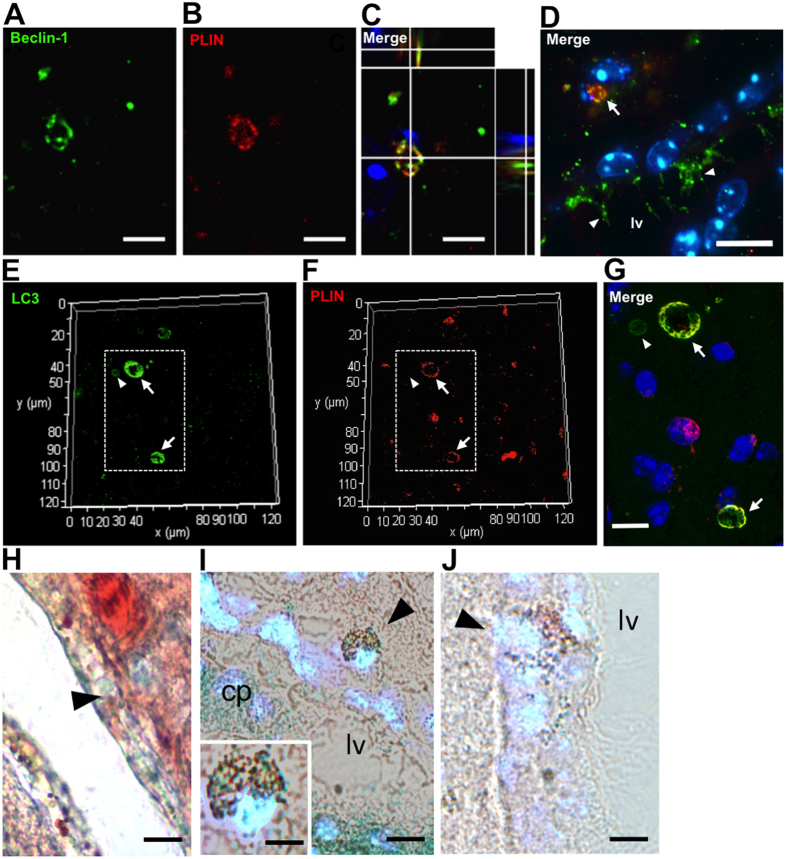
Expression of Beclin-1, LC3 or staining for SA-βgal in LLC of the brain lateral ventricle walls of middle-aged and old mice. (**A–D**) Representative fluorescence photomicrographs showing co-localization of Beclin-1 with PLIN in LLC in the ventricular medial wall area of middle-aged mice. (**D**) Beclin-1^+^ cilia in ependymal cells are indicated by white arrowheads. Cell nuclei were counterstained with Hoechst. Beclin1^+^PLIN^+^ LLC is indicated (white arrow) (**E–G**) Representative fluorescence photomicrographs showing co-localization of LC3 with PLIN in LLC in the ventricular lateral wall area of old mice (white arrows). Dotted lines in (**E**,**F**) correspond to merged area shown in higher magnification (**G**). LC3^+^ autophagosome in a cell without lipid accumulation is indicated by white arrowheads. (**H,I**) Bright field photomicrograph of Oil Red-O^+^ SA-βgal^+^ LLC (black arrowheads) in the ventricular lateral wall merged with fluorescent Hoechst counterstaining in old and middle-aged mice, respectively. (**J**) Photomicrograph of Oil Red-O^+^ SA-βgal^−^ LLC (black arrowhead) in the ventricular lateral wall merged with fluorescent Hoechst counterstaining. cp: choroid plexus; lv: lateral ventricle. Scale bars: 2.5 μm (**A–C**); 10 μm (**D–J**).

Considering that lipid droplets have been associated with autophagosome formation in stress conditions^28^, we further evaluated whether autophagy could be occurring in cerebral LLC. We analyzed the expression of Beclin-1, which is a key molecule involved in autophagosome formation (Fig. 4A–D). We found LLC with large lipid bodies co-expressing PLIN, a lipid droplet-associated protein, and Beclin-1 in the lateral ventricle medial wall (Fig. 4A–D), thus suggesting the existence of “autolipophagosomes” in these cells. In addition, we observed staining for Beclin-1 in the cilia of PLIN^−^ ependymal cells in aged mice (Fig. 4D). Moreover, we verified co-expression of PLIN and LC3, which is an autophagolysosome marker, in LLC in the ventricular lateral wall (Fig. 4E–G). Autophagy as a response to age-related stress could lead to cell senescence. In this context, we found some Oil Red O^+^ LLC in the lateral subventricular and cortical regions of aging mice with senescence associated β-galactosidase (SA-β-gal) staining (Fig. 4H,I), which correspond to one of the main biomarkers for senescent cells^29^. However, Oil Red O^+^ LLC without staining for SA-β-gal were also identified in the lateral subventriclular and cortical regions in old mice (Fig. 4J). Senescent cells can release inflammatory cytokines, which can have paracrine effects in non-senescent neighbor cells, for instance, contributing to increased local oxidative stress, basal inflammation and tissue degeneration^30^.

**Figure 5:**
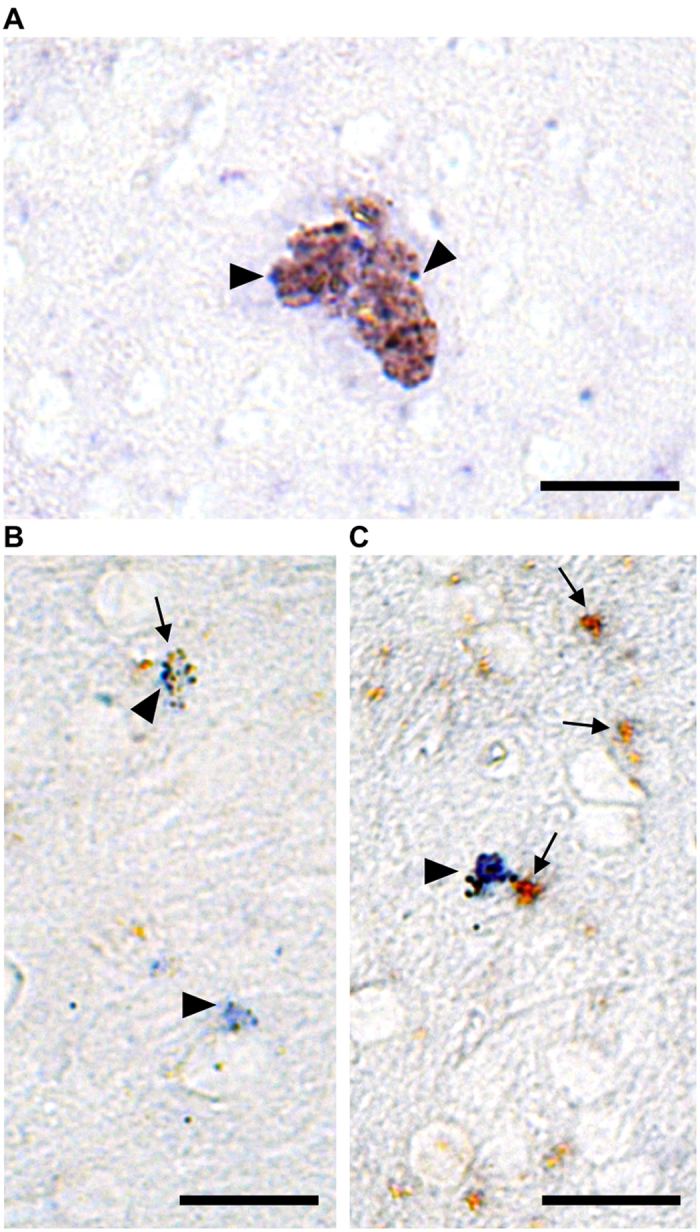
LLC produce TNF-α mRNA in the old mouse brain. (**A–C**) Bright field photomicrograph shows brownish PLIN^+^ LLC expressing TNF-α mRNA (blue) in the cerebral cortex. *In situ* hybridization for TNF-α mRNA, positively detected using alkaline phosphatase (arrowheads indicate blue staining), was done in combination with immunoperoxidase for PLIN (arrows indicate brown staining) in old brain tissue. Scale bars: 20 μm.

Next, we sought to determine whether cerebral LLC could produce pro-inflammatory factors, more specifically, TNF-α transcripts. We combined *in situ* hybridization for detection of TNF-α mRNA with immunoperoxidase to identify cellular PLIN expression. We found TNF-α transcripts in various regions of the brain, including cortex, striatum, ventricular/subventricular region and pia mater (Supplementary Fig. S2), thus confirming the association of LLC with the basal inflammatory state of the aging brain. However, we found PLIN^+^ LLC expressing TNF-α mRNA mainly in the brain cortical region (Fig. 5A–C), thus suggesting that LLC might actively participate in the neuroinflammatory processes in the brain aging.

### LLC correspond to distinct cell types accumulating lipids in the aged brain

**Figure 6:**
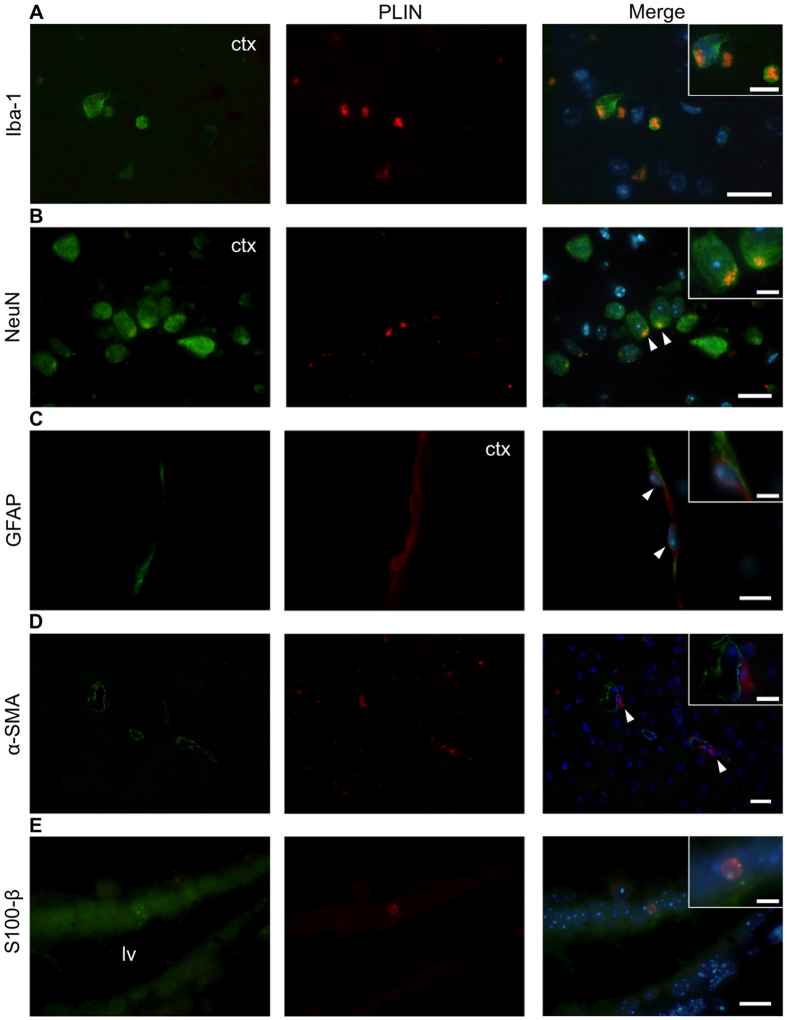
Cellular phenotypes of LLC in the middle-aged mouse brain. (**A**) PLIN^+^ Iba-1^+^ microglial cells. (**B**) PLIN^+^ NeuN^+^ neurons. (**C**) PLIN^+^ GFAP^+^ glia limitans astrocytes. (**D**) PLIN^+^ cells adjacent to α-SMA^+^ pericytes (white arrowheads) in the brain cortical region. (**E**) PLIN^+^ S100β^+^ ependymal cells in the medial lateral ventricle wall (white arrowheads). Brain tissue samples were analyzed by immunohistochemistry using the lipid droplet specific marker Perilipin (PLIN, in red) in combination with cellular phenotype markers indicated in the figure (in green). Insets show higher magnification of distinct PLIN^+^ cells. Nuclei were stained with Hoechst (in blue). Bars: 20 μm. Inset bars: 10 μm.

Given that LLC are widely and differentially distributed in the aging brain and show distinct morphologies, we decided to evaluate their phenotype performing *in situ* immunofluorescence using PLIN in combination with specific markers for glial cells, neurons or myofibroblastic progenitor cells. Our data show brain cortical PLIN^+^ cells express distinct phenotype markers. Iba1^+^ microglial cells showed a lipid-laden multilocular pattern (Fig. 6A). In addition, we observed NeuN^+^ neurons (Fig. 6B) and GFAP^+^ astrocytes, more specifically, glia limitans (Fig. 6C), expressing PLIN in the cortical region of the aged brain. On the other hand, αSMA^+^ pericytes that did not show PLIN expression were located adjacent to PLIN^+^ LLC cells in the cortical region (Fig. 6D). Moreover, we found PLIN^+^S100β^+^ ependymal cells containing larger lipid droplets in the lateral ventricle walls (Fig. 6E). Conjointly, these data reveal that LLC correspond to distinct cellular phenotypes in the aging brain.

## Discussion

Our findings reveal that the density of cerebral LLC increases in distinct regions of the aging brain and that these cells can have autophagic and SA-β galactosidase activity as well as express mRNA for TNF-α pro-inflammatory cytokine, thus suggesting their participation in the age-associated neurodegenerative process. Cerebral LLC presenting distinct phenotypes indicate that these cells, derived from bone marrow/mesoderm or neural crest/neuroectoderm layers, can accumulate lipids in the aging brain. Below, we discuss the origins of cerebral LLC and their relation with autophagy, senescence and inflammation in the aging brain.

LLC in the meningeal connective tissue and in the nervous tissue may include lipid-laden microglial cells. In the current study, we found that Iba1^+^ microglia expressing perilipin is in association with the expression of TNF-α transcripts in the aged brain. In this context, it is interesting to note that previous findings suggest that the activation of microglia by TNF-α stimulates phagocytosis of oxidized lipids of myelin sheaths and that such lipid laden microglia are prominent in the lesions of patients with multiple sclerosis^31^,^32^. However, further work will be required to elucidate the phenotypes and functional roles for lipid-laden microglia in the normal aging brain. Indeed, microglial cells can also secrete anti-inflammatory cytokines and may play roles in adaptive processes including tissue repair^33^.

Mesenchymal cells that derive from the neural crest are found in the pia mater and perivascular areas in the cerebral cortex and striatum^26^. These cells share, along with pericytes and mesenchymal cells derived from the mesoderm, the potential to differentiate into lipoblasts and adipocytes as well as smooth muscle cells, chondroblasts and/or other specialized connective tissue cell lineages, under specific culture conditions^34^. Although we did not find PLIN^+^ SMA^+^ pericytes cells around vessels in the brain, we do not discard the possibility that neural crest-derived meningeal mesenchymal cells can acquire the capacity of accumulating lipids with age.

Our observation that Oil Red O^+^ cells were found in the pia mater and around blood vessels in the brain suggest to us that LLC may migrate from the adjacent connective tissue and perivascular space into the neural parenchyma in the aging brain. In this sense, it is known that immune cells enter the brain tissue in a number of pathologies, including Alzheimer’s disease^35^ and Parkinson’s disease^36^. Moreover, we showed that the frequency of LLC deeper in the neural parenchyma increases in relation to LLC around blood vessels in the brain tissue between middle age and old age.

We also observed some GFAP^+^ glia limitans externa accumulating lipids. These cells, as neurons, oligodendrocytes and ependymal cells, derive from the neuroectoderm. Glia limitans externa, located in between the brain cortical parenchyma and pia mater, make contact with meningeal cells and are known to participate in the brain-CSF barrier^37^. We failed to find PLIN^+^GFAP^+^ astrocytes in the brain parenchyma of aged mice. However, another group has shown that these cells can accumulate lipids in the aging human optic nerve^38^.

Using an antibody against PLIN as one method to identify lipid droplets in different cell types, we found that PLIN^+^ LLC were considerably fewer than the number of LLC that stained with Oil Red O, indicating that there are many LLC that are not detected with the anti-PLIN antibody. Furthermore, we found that most Oil Red O^+^ LLC in brain tissue sections do not exhibit autofluorescence (Supplementary Fig. S1) indicating that the Oil Red O staining in LLC is not lipofuscin^39^. Other groups have also shown that PLIN^+^ lipid droplets or LAMP1^+^ lysosomes in the retina do not correspond to lipofuscin^40^,^41^. On the other hand, we cannot rule out the possibility that some brain cells do contain both lipofuscin and lipid droplets. In contrast to neurons in the brains of rats and mice, which exhibit little or no lipofuscin accumulation during aging, lipofuscin deposition in some neurons during the aging process is common in humans^14^,^39^. Moreover, lipid droplets have been reported to be present in association with amyloid-β peptide-positive neurons in Alzheimer’s disease patients^42^.

The presence of Oil Red O^+^ and PLIN^+^S100β^+^ LLC in the lateral ventricles indicate that ependymal cells may accumulate lipids with age. These data are in agreement with previous studies in which LLC were detected in the lateral ventricles of middle-aged and old mice^23^,^24^. In addition, we verified an asymmetric distribution of LLC in the lateral ventricle walls of old mice, particularly among the dorsal, medial and ventral subregions; and in the lateral wall compared to the medial wall. The subventricular region of the lateral wall is where adult neurogenesis occurs^43^, suggesting a potential role for LLC in the decline of neurogenesis that occurs during aging^44^,^45^,^46^. We also found LLC in the olfactory bulb, to where subventricular zone neural precursor cells migrate and complete their differentiation^47^. The reason for the asymmetric distribution of LLC in the lateral ventricle walls is unknown. Possibly, intrinsic differences between cells in different domains within the lateral ventricle walls could make cells in some areas more prone to lipid accumulation. In fact, distinct neurogenic subregions in the medial and lateral walls were recently described^48^.

We found that LLC in the lateral subventricular zone of the aged brain express the positive autophagy regulator Beclin-1 and LC3, a marker for the fusion of autophagosomes with lysosomes^49^. It is known that age is associated with a decrease in autophagy, which could contribute for the onset and progression of neurodegenerative diseases^50^. Our data showing Beclin-1 or LC3 expression in PLIN^+^ lipid droplets of LLC in the subventricular zone suggest that these cells could have lipophagic activity^51^, possibly in order to regulate intracellular lipid stores and prevent and rescue cells from lipotoxic damage resulting from oxidative stress and inflammation in the aging brain^49^. Alternatively, LLC with multilocular morphology could represent a population of cells with blocked autophagy. Indeed, while lipids can normally activate autophagy, this same process can be blocked by higher concentrations of lipids^28^. We also found that Beclin-1 was observed in ependymal cells that exhibited a senescence-related phenotype as indicated by SA-β galactosidase staining. Changes in the secretome of LLC could lead to the formation of senescence-associated secretory phenotype (SASP)^52^ and contribute to neuroinflammation and oxidative stress. In this regard, aged ependymal cells exhibit cilia-deficient patches^24^ and autophagy induction has been related to ciliogenesis in mammalian cells in other tissues^53^. Possibly, autophagosome formation in the aged ependymal cells could indicate that these cells are trying to remove damaged cilia.

Age-associated oxidative stress due to decreased antioxidant enzyme activity and pro-inflammatory signaling mediated by TNF-α/NFκB can impair autophagy^54^. In the current study, we found that at least some PLIN^+^ LLC express TNF-α mRNA. In the aging brain, microglial cells express TNF-α^55^. Moreover, neural precursor cells express TNF-α and its receptor^56^. In agreement with these data, Tha *et al*.^57^ have shown increased levels of pro-inflammatory cytokines, including TNF-α, in the brain of the senescence accelerated mouse. In this context, TNF-α has also been considered a potent suppressor of adult neurogenesis^58^ and a stimulator of cellular stress which leads to cell damage, reduced tissue repair and increased tissue dysfunction, which may contribute to neurodegenerative diseases associated with aging^59^. In conclusion, our data suggest that LLC accumulate in the aging mouse brain, with certain brain regions exhibiting more LLC than others. LLC may have impaired autophagy, and pro-inflammatory and/or senescent phenotypes, which could contribute to deficits in neurogenesis and synaptic plasticity that can occur in normal aging. Because aging is the major risk factor for Alzheimer’s and Parkinson’s diseases, it will be of considerable interest to elucidate possible roles for LLC in these disorders.

## Methods

### Animals

Balb/c mice were kept under a constant light-dark cycle (12 h) and temperature of 25°C, with standard food and water *ad libitum*. Female young (3 months-old), middle-aged (12 months-old) and old (18–22 months old) animal groups, used in this study, were described as equivalent to young (20–30 years old) middle age (38–47 years old) and Old (58–69 years old) human being age ranges^60^. All protocols and handling of the subjects were carried out under the approval of the Ethics Committee for Animal Experimentation at Federal University of Rio de Janeiro. Methods were carried out in accordance with the approved guidelines.

### Histological analyses for LLC identification and quantification

Brain samples were obtained after transcardial perfusion with phosphate buffered saline (PBS) and heparin, followed by 4% paraformaldehyde (Sigma-Aldrich). Brains were removed, post-fixed overnight in 4% paraformaldehyde solution and treated with 30% sucrose for cryoprotection. Tissues were embedded in Tissue Tek resin (Sakura). Cryostat 5 μm-thick tissue sections between bregma +0.5 and +0.26 were prepared (n = 3, per animal). An interval of at least 15 μm was maintained between the brain tissue sections collected.

Brain cryosections were washed in PBS and 100% propylene glycol (Sigma-Aldrich) before incubation in 7% Oil Red-O dye (Sigma-Aldrich) for 2 h. Tissue sections were washed in 85% propylene glycol and water before counter-staining with toluidine blue dye. Slides with tissue samples were mounted in an aqueous medium consisting of 50% glycerol (Sigma-Aldrich) and 50%, PBS pH 8.5. For broad-spectrum autofluorescence analyses characteristic of lipofuscin pigments, brain tissue sections were mounted in aqueous medium and photographed under 488 nm UV excitation after which the slides were unmounted and then used for Oil Red-O staining. Tissue photomicrographs were taken under the light of an Axioplan microscope (Zeiss) using a 5.0 Megapixels camera (Evolution).

The quantification of Oil Red O^+^ LLC was performed in the cortex, striatum and pia mater. In the cortex, cell counts included the primary somatosensory cortex, the primary and secondary motor cortices and the cingulum. In the pia mater, cells were counted throughout the perimeter of the entire brain tissue section. For our quantification purposes, two or more Oil Red-O^+^ lipid droplets associated with a cell containing a single clearly identifiable toluidine blue^+^ nucleus was considered as one LLC. The density corresponding to the quantification of LLC per area was performed by dividing the number of cells in each brain region by its respective area (for cortex and striatum) or perimeter (for pia mater), which were calculated using ImageJ software (Rasband, W.S., U. S. National Institutes of Health, Bethesda, Maryland, USA, http://rsb.info.nih.gov/ij/, 1997–2012). To evaluate Oil Red O staining around the lateral ventricles, densitometric analyses were performed. The area and intensity of Oil Red O (red signals) were determined using Adobe Photoshop software’s (Adobe Systems, Inc.) histogram analyses and normalized by the lateral ventricle perimeter in each image. Oil Red O staining intensity was quantified in three distinct subregions of the lateral ventricle (dorsal, medial and ventral) within 20 μm depth from the ventricular wall. Dorsal subregion: a dorsoventral length average of 205.1 ± 11.4μm starting from the lateral corner was analyzed for all age groups; ventral subregion: a ventrodorsal length average of 295.7 ± 15.1μm starting from the ventral extremity of the lateral ventricles was analyzed for all groups. The medial subregion of the lateral ventricle consisted in the area between the dorsal and ventral subregions.

### Senescence-associated β-galactosidase staining

For the detection of galactosidase (SA-β-gal) activity, brain tissue sections previously stained with Oil Red-O, as described above, were processed using the Senescent Cells Histochemical Staining Kit (Sigma) according to manufacturer’s guidelines. Bright field photomicrographs of SA-β-gal staining and photomicrographs of cellular nuclei stained with Hoechst (Sigma-Aldrich; 861405) were obtained using an Olympus LX-17 fluorescence microscope.

### Immunohistochemistry

Paraffin embedded 5 μm-thick coronal brain tissue sections were deparaffinized and subjected to heat-mediated antigen retrieval in pH 6.0 sodium citrate buffer. Unspecific epitopes were blocked with 10% normal goat serum following incubation with the primary monoclonal rabbit anti- C-terminus residues of human S100 beta antibody (Clone EP1576Y; Abcam; ab52642) or the following polyclonal unconjugated antibodies that also react with mouse tissue: rabbit anti-Iba1 carboxy terminal sequence (Wako; 019–19741); rabbit anti-human recombinant GFAP (Abcam; ab7260); rabbit anti-N terminus of alpha smooth muscle actin (human) synthetic peptide (Abcam; ab5694), rabbit anti-human beclin-1 synthetic peptide (Abcam; ab62472); or goat anti-human PLIN (N-14) (PLIN) (Santa Cruz, sc-47322) for 16 hours at 4 °C. Tissue sections were then washed in PBS and incubated with the secondary antibodies donkey anti-rabbit alexa 488 or donkey anti-goat alexa 555 (Invitrogen) for 2 h at 37 °C before washing in PBS and staining with Hoechst 1 μg/ml. Slides with tissue samples were mounted using SHUR/Mount medium (Ted Pella Inc.). Photomicrographs were obtained using an Olympus LX-17 fluorescence microscope.

For immunoperoxidase, samples were washed in PBS and tissue peroxidase was blocked by incubation with 0.03% hydrogen peroxidase in PBS for 30 minutes in the dark. Tissue samples were washed in PBS (5 × 5 min, each) and incubated with blocking buffer for 1 h before adding goat anti-human PLIN antibody (N- 14) diluted 1:100 in blocking buffer. Following an overnight incubation at 4 °C, samples were washed and incubated with donkey anti-goat antibody conjugated to horseradish peroxidase (HRP) for two hs at RT. After washing, HRP reaction was performed by incubation with 0.03% hydrogen peroxide in diaminobenzidine (DAB) solution (75 μg/ml in PBS) for 10 min. Samples were washed in PBS, dehydrated in progressively higher concentrations of ethanol, cleared in xylol and mounted in Entellan (Merck).

### TNF-α probe synthesis and *in situ* hybridrization

The probe sequence of TNF-α was generated by polymerase chain reaction (PCR) using the primers 5′-GAG CCT TTC TGC AAA GGG AG-3′ (sense) and 5′-GAG GAG GCA GCA AAA AAC AG -3′ (antisense). The cDNA template obtained from aspirated bone marrow of adult mice was isolated using TRIzol reagent (Life Technologies) and synthesized using M-MLV reverse transcriptase (Promega). The PCR result showed a well-defined band of 332 bp after 30 cycles with annealing temperature at 60°C. Plasmids were generated from the PCR product using the pCRII-TOPO^®^kit (Life Technologies) and inserted into thermocompetent DH5α *Escherichia coli* (Life Technologies). Plasmids from different colonies were isolated using the Plasmid NucleoSpin kit (Macherey-Nagel) and the sequence and orientation of inserts were verified using Sanger sequencing. Plasmids containing TNF-α sequence were linearized with Xbal enzyme (Thermo Scientific) for 4 hours and purified by precipitation with sodium acetate. The synthesis of probes was performed with SP6-riboprobe System kit (Promega). RNA probes were purified by precipitation with lithium chloride (Sigma-Aldrich). *In situ* hybridization was performed according to Charrier *et al*.^61^ and revealed with anti-DIG Fab fragments conjugated to alkaline phosphatase (Roche) diluted 1: 2000. Subsequently, brain tissue sections were incubated in the presence of immunoperoxidase to detect PLIN in cells expressing TNF-α mRNA, as described above.

### Statistics

One-way ANOVA analyses were performed using the GraphPad Prism Software (GraphPad Software). Results comparing distinct age groups were considered statistically significant when p < 0.05.

## Additional Information

**How to cite this article**: Shimabukuro, M. K. *et al*. Lipid-laden cells differentially distributed in the aging brain are functionally active and correspond to distinct phenotypes. *Sci. Rep.* **6**, 23795; doi: 10.1038/srep23795 (2016).

## Supplementary Material

Supplementary Information

## Figures and Tables

**Table 1:** Age-related ratio of Oil Red O staining between the lateral and medial walls of the lateral ventricles.

Groups	Lateral wall: Medial wall
Middle-Aged	2.1: 1
Old	1.2: 1

Data expressed as mean ± SEM. N = 3 mice per group.

**Table 2:** Age-related changes in the ratio of BV-LLC^a^ versus P- LLC^b^ in the cortex and striatum.

Group	Cortex	Striatum
	BV-LLC: P-LLC	BV-LLC: P-LLC
Middle-aged	4: 1	7.5: 1
Old	2.1: 1	5.9: 1

^a^BV-LLC: Perivascular lipid laden cells.

^b^P-LLC: Parenchymal lipid laden cells. Data expressed as mean ± SEM. N = 3 mice per group.
