# Supplementary material for: Lipid-laden cells differentially distributed in the aging brain are functionally active and correspond to distinct phenotypes
Source: Sci Rep. 2016 Mar 31;6:23795. doi: 10.1038/srep23795 (PMC4814830; doi:10.1038/srep23795)
Supplement: Supplementary Information [file srep23795-s1.pdf]

Lipid-laden cells differentially distributed in the aging brain are functionally active and correspond to distinct phenotypes.

Marilia Kimie Shimabukuro; Larissa Gutman Paranhos Langhi; Ingrid Cordeiro; José M. Brito; Claudia Maria de Castro Batista; Mark P. Mattson; Valeria de Mello Coelho.

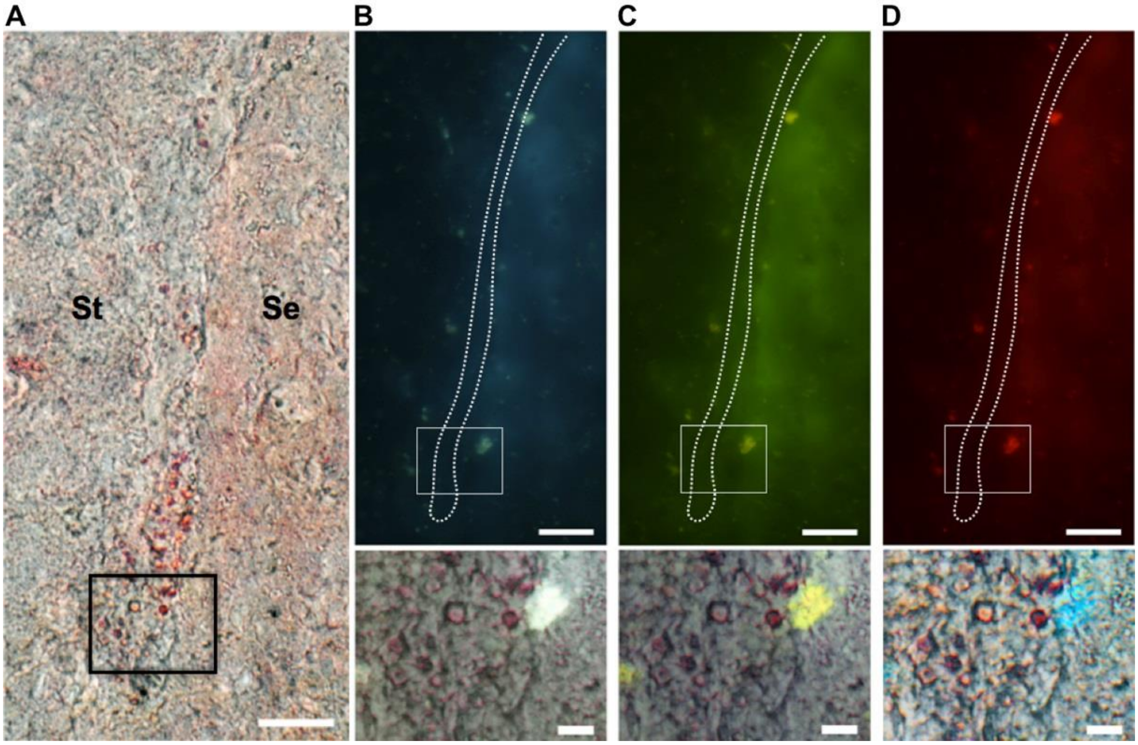

Supplementary Fig. S1. Oil Red-O^{+} lipid droplets did not co-localize with autofluorescent signals in brain tissue. A. Representative photomicrograph showing the ventral portion of the lateral ventricle after Oil Red-O staining. **B-D.** Fluorescence photomicrographs of the same area in (A) showing autofluorescence signals in three different spectra detected before the Oil Red-O staining procedure. In A-D, the black and white defined areas correspond to the same region in the lower panels in (B-D), where Oil Red-O staining is merged with autofluorescence (lipofuscin). In B-D the dotted white lines delineate the lateral ventricle of aged mice. Scale bars: 20 µm (A-D); 5 µm (B, C, D insets).

Lipid-laden cells differentially distributed in the aging brain are functionally active and correspond to distinct phenotypes.

Marilia Kimie Shimabukuro; Larissa Gutman Paranhos Langhi; Ingrid Cordeiro; José M. Brito; Claudia Maria de Castro Batista; Mark P. Mattson; Valeria de Mello Coelho.

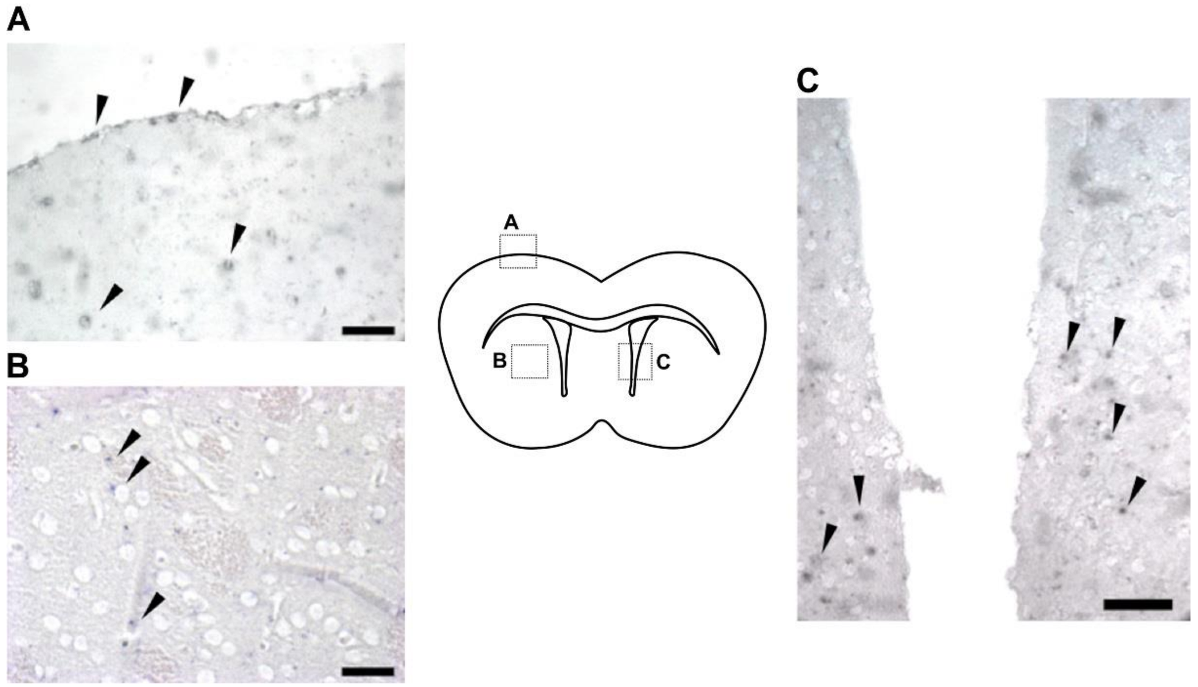

Supplementary Fig. S2. TNF-α mRNA expression in the old mouse brain. A-C. Representative bright field photomicrographs of TNF-α mRNA analyzed by *in situ* hybridization. Positive signals corresponding to TNF-α transcripts are shown in brain cortex and pia mater (A), striatum (B) and lateral ventricle walls (C). Arrowheads indicate some positive signals for TNF-α mRNA. Scale bars: 50 μm.
